# Supplementary material for: A Catalogue of Structural Variation across Ancestrally Diverse Asian Genomes
Source: Nat Commun. 2024 Nov 4;15:9507. doi: 10.1038/s41467-024-53620-8 (PMC11535549; doi:10.1038/s41467-024-53620-8)
Supplement: Supplementary file 1 — Supplementary Information [file 41467_2024_53620_MOESM1_ESM.pdf]

Supplementary Information

A Catalogue of Structural Variation across Ancestrally Diverse Asian Genomes

Joanna Hui Juan Tan[1,*], Zhihui Li[1,*], Mar Gonzalez Porta[1,2,*], Ramesh Rajaby[1,3,*], Weng Khong Lim[1,4,5,6], Ye An Tan[7], Rodrigo Toro Jimenez[1], Renyi Teo[1], Maxime Hebrard[1], Jack Ling Ow[1], Shimin Ang[1], Justin Jeyakani[1], Yap Seng Chong[8,9], Tock Han Lim[10], Liuh Ling Goh[11], Yih Chung Tham[12,13], Khai Pang Leong[11], Calvin Woon Loong Chin[14,15], SG10K_Health Consortium[^], Sonia Davila[6,16,17, 18], Neerja Karnani[19,20,21], Ching-Yu Cheng[12,22], John Chambers[23,24,25], E Shyong Tai[4,26,27,25], Jianjun Liu[28,29], Xueling Sim[7], Wing Kin Sung[1,30,31], Shyam Prabhakar[1,32,33,#], Patrick Tan[1,4,5,25,#], Nicolas Bertin[1,#]

Table of Contents

Supplementary Notes......2

Supplementary Note 1: Benchmarking of SV calling pipeline for deletions and insertions......2

Supplementary Note 2: Benchmarking of SV calling pipeline for duplications......6

Supplementary Note 3: Identifying novel variants with respect to gnomAD-SV catalogue......7

Supplementary Note 4: Identifying novel variants with respect to 1000G-SV catalogue......8

Supplementary Figures......9

Supplementary Fig. 1 Different types of structural variations detected in SG10K-SV.......9

Supplementary Fig. 2 Benchmarking of various SV callers for deletions and insertions.......10

Supplementary Fig. 3 True positive, false positive and false negative counts for Manta, Delly, Smoove and their combination for all classed of SVs......11

Supplementary Fig. 4 Comparison of SurVindel2 and Manta-SVimmer-Graphtyper2 pipeline for duplication identification.......12

Supplementary Fig. 5 Barplot showing the number of duplications detected by Manta-SVimmer-Graphtyper2 and SurVindel2 in different genomic regions.......13

Supplementary Fig. 6 Violin plot showing the number of events per genome for the Validation datasets.......14

Supplementary Fig. 7 Allele distribution for the two validation datasets.......15

Supplementary Fig. 8 Samplot of a 9.16kb deletion event overlapping the *PRKAG2* gene region.......16

Supplementary Fig. 9: Distribution of novel Asian-specific and known Asian-specific SVs across different allele frequency bins 17

Supplementary Fig. 10 Scatter plot of the top-2 principal components of a SG10K_Health dataset Single Nucleotide Variant based PCA analysis showing the population structure in the Singaporean population 18

Supplementary Fig. 11 PCA of variants in the discovery dataset showing the population structure in the SG10K-SV-r1.4. 19

Supplementary Fig. 12 Distribution of SVs shared among ethnic group across different allele frequency bins. 20

Supplementary references ***21***

SG10K_Health Consortium ***22***

Supplementary Notes

Supplementary Note 1: Benchmarking of SV calling pipeline for deletions and insertions

We aimed to comprehensively assess the performance of our SV calling pipeline by comparing the SG10K-SV (Manta[1]) pipeline with three other popular SV detection algorithms such as Delly[2] and Smoove[3].

To accurately benchmark the performance of our SV detecting pipeline, we downloaded a subset of 34 1000 Genome samples with both long and short read whole genome sequencing (WGS) data. We retrieved the 30x short read WGS CRAM files from https://registry.opendata.aws/1000-genomes/. Long-read sequencing data have become the technique of choice for SV detections and hence it will serve as the truth set for the comparison. We retrieved the comprehensive catalogue of SVs detected using long-read sequencing from Ebert *et al.*[4] (https://ftp.1000genomes.ebi.ac.uk/vol1/ftp/data_collections/HGSVC2/release/v2.0/integrated_callset/) to ascertain the sensitivity and precision of the short-read SVs predicted using our SV detection pipeline and 2 other SV detection algorithms.

<u>SV discovery using Manta</u>

Manta[1] was executed in the single sample mode to identify deletions and insertions in the 34 1000G samples using default parameters. We used SVimmer[5] to cluster SVs across samples using the default parameters and re-genotyped the SVs in each sample using Graphtyper2[6] with default parameters. We then merged the individual re-genotyped VCF using Graphtyper2's *Vcfmerge* function. Lastly, we retained PASS calls made under the aggregated genotyping model for downstream analysis. In addition, we applied additional filters recommended by Graphtyper2.

For deletions, we filter the variants using bcftools with the following command:

```
bcftools 'INFO/SVMODEL="AGGREGATED" && FILTER="PASS" && SVTYPE="DEL" && QD > 9  &&
(ABHet > 0.3 || ABHet < 0 ) && (AC/NUM_MERGED_SVS) < 25  && PASS_AC > 0 &&
PASS_ratio > 0.1' ${vcf} | bcftools filter -i 'FMT/FT ="PASS" ' --set-GTs . - |
bcftools view -c1 -s ${meta} --output ${prefix}.DEL.vcf.gz -Oz --threads $task.cpus
-
```

For duplications, we retain variants which passed the following criteria:

```
bcftools view -i 'INFO/SVMODEL="AGGREGATED" && FILTER="PASS" && SVTYPE="DUP" &&
QD > 5 && (AC/NUM_MERGED_SVS) < 25 && PASS_AC >0 ' ${vcf} |
bcftools filter -i 'FMT/FT ="PASS" ' --set-GTs . - | bcftools view -c1 -s ${meta} -
-output ${prefix}.DUPonly.vcf.gz -Oz --threads $task.cpus -
```

Lastly, for insertions, we filtered the variants with bcftools using the following command:

```
bcftools view -i 'INFO/SVMODEL="AGGREGATED" && FILTER="PASS" && SVTYPE="INS" &&
PASS_AC >0 && (AC/NUM_MERGED_SVS) < 25 && PASS_ratio > 0.1 && (ABHet > 0.25 ||
ABHet < 0) && MaxAAS > 4' ${vcf} | bcftools filter -i 'FMT/FT ="PASS" ' --set-GTs .
--threads $task.cpus - | bcftools view -c1 -s ${meta} --output
${prefix}.INSonly.vcf.gz -Oz --threads $task.cpus -
```

SV discovery using Delly

Delly[2] v1.2.6 was executed in the single sample mode to identify deletions and insertions in the 34 1000G samples using default parameters. BCFtools[7] was used to convert the bcf output from Delly to VCF format before clustering SVs across samples using SVimmer[5]. The SVs were re-genotyped in each sample using Graphtyper2[6] with default parameters. We merged the individual re-genotyped VCF using Graphtyper2's *Vcfmerge* function Lastly, we retained PASS calls made under the aggregated genotyping model for downstream analysis. In addition, we applied additional filters recommended by Graphtyper2.

For deletions, we filter the variants using bcftools with the following command:

```
bcftools 'INFO/SVMODEL="AGGREGATED" && FILTER="PASS" && SVTYPE="DEL" && QD > 9  &&
(ABHet > 0.3 || ABHet < 0 ) && (AC/NUM_MERGED_SVS) < 25  && PASS_AC > 0 &&
PASS_ratio > 0.1' ${vcf} | bcftools filter -i 'FMT/FT ="PASS" ' --set-GTs . - |
bcftools view -c1 -s ${meta} --output ${prefix}.DEL.vcf.gz -Oz --threads $task.cpus
-
```

For duplications, we retain variants which passed the following criteria:

```
bcftools view -i 'INFO/SVMODEL="AGGREGATED" && FILTER="PASS" && SVTYPE="DUP" &&
QD > 5 && (AC/NUM_MERGED_SVS) < 25 && PASS_AC >0 ' ${vcf} |
bcftools filter -i 'FMT/FT ="PASS" ' --set-GTs . - | bcftools view -c1 -s ${meta} -
-output ${prefix}.DUPonly.vcf.gz -Oz --threads $task.cpus -
```

Lastly, for insertions, we filtered the variants with bcftools using the following command:

```
bcftools view -i 'INFO/SVMODEL="AGGREGATED" && FILTER="PASS" && SVTYPE="INS" &&
PASS_AC >0 && (AC/NUM_MERGED_SVS) < 25 && PASS_ratio > 0.1 && (ABHet > 0.25 ||
ABHet < 0) && MaxAAS > 4' ${vcf} | bcftools filter -i 'FMT/FT ="PASS" ' --set-GTs .
--threads $task.cpus - | bcftools view -c1 -s ${meta} --output
${prefix}.INSonly.vcf.gz -Oz --threads $task.cpus -
```

SV discovery using Smoove

Smoove was executed in the single sample mode to identify structural variations in the 34 1000G samples using Smoove Call function with default parameters. Variants were merged across samples using the Smoove Merge function with default parameters. Lastly, SVs were re-genotyped in each sample using the Smoove Genotype function with default parameters.

Combining SVs detected across the three algorithms

We obtained the single sample calls from each of the algorithms (Manta, Smoove, Delly) and clustered across all samples and algorithm using SVimmer[5] with the default parameters. Lastly, we re-genotyped SVs in each sample using Graphtyper2[6] with default parameters and merged the individual re-genotyped VCF using Graphtyper2's *Vcfmerge* function Lastly, we retained PASS calls made under the aggregated genotyping model for downstream analysis. In addition, we applied additional filters recommended by Graphtyper2.

For deletions, we filter the variants using bcftools with the following command:

```
bcftools 'INFO/SVMODEL="AGGREGATED" && FILTER="PASS" && SVTYPE="DEL" && QD > 9  &&
(ABHet > 0.3 || ABHet < 0 ) && (AC/NUM_MERGED_SVS) < 25  && PASS_AC > 0 &&
PASS_ratio > 0.1' ${vcf} | bcftools filter -i 'FMT/FT ="PASS" ' --set-GTs . - |
bcftools view -c1 -s ${meta} --output ${prefix}.DEL.vcf.gz -Oz --threads $task.cpus
-
```

For duplications, we retain variants which passed the following criteria:

```
bcftools view -i 'INFO/SVMODEL="AGGREGATED" && FILTER="PASS" && SVTYPE="DUP" &&
QD > 5 && (AC/NUM_MERGED_SVS) < 25 && PASS_AC >0 ' ${vcf} |
bcftools filter -i 'FMT/FT ="PASS" ' --set-GTs . - | bcftools view -c1 -s ${meta} -
-output ${prefix}.DUPonly.vcf.gz -Oz --threads $task.cpus -
```

Lastly, for insertions, we filtered the variants with bcftools using the following command:

```
bcftools view -i 'INFO/SVMODEL="AGGREGATED" && FILTER="PASS" && SVTYPE="INS" &&
PASS_AC >0 && (AC/NUM_MERGED_SVS) < 25 && PASS_ratio > 0.1 && (ABHet > 0.25 ||
ABHet < 0) && MaxAAS > 4' ${vcf} | bcftools filter -i 'FMT/FT ="PASS" ' --set-GTs .
--threads $task.cpus - | bcftools view -c1 -s ${meta} --output
${prefix}.INSonly.vcf.gz -Oz --threads $task.cpus -
```

Calculating precision, recall and F1-Score

To evaluate the performance of different SV algorithm, we focus the test on the presence and absence of the variants in the long read dataset. We calculate the precision, recall and F1-Score using Truvari[8] with the SV calls from long read data

from Ebert *et al.*[4] as the truth set. A variant is defined as a true positive (TP) if the variant is found in both short-read and long-read dataset. A variant is defined as a false positive (FP) if it is not found in the long read dataset.

Precision is defined as:

$$Precision = \frac{TP}{TP+FP} \quad (1)$$

Recall is defined as:

$$Recall = \frac{TP}{TP+FN} \quad (2)$$

F1-score is defined as:

$$F1 = 2 * \frac{Precision*Recall}{Precision+Recall} \quad (3)$$

<u>Evaluation of SV detection pipelines using 34 1000G project WGS data</u>

The precision and recall for SV detection varied depending on the method. Fig 1c, d and e show the precision, recall and F1 (combined statistics of precision and recall) of the three different SV calling pipeline for calling structural variations. Delly+Graphtyper2 has the highest precision in terms of SV detection for both 30x and 15x sequencing libraries. However, Manta+Graphtyper2 has a higher recall and F1 score compared to the rest of the pipelines. Combining SVs detected by all three pipelines did not improve the precision, recall and F1-Score compared to running the Manta+Graphtyper2 pipeline.

When analyzing the SVs separately based on the SV classes, Delly+Graphtyper2 has the highest precision in terms of deletion and insertion detection for both 30x and 15x sequencing libraries (Supplementary Fig. 2). In terms of recall and F1-score, Manta+Graphtyper2 outperforms the other pipelines for both deletions and insertions.

Next, we evaluate how sequencing read depth affects the performance of the SV pipelines. We down-sampled the 30x CRAM files to 15x using Sambamba[9] and we evaluate the performance of the four different approaches to detect SVs. Differences

in sequencing depth affect precision and recall (Fig. 1c, d, e, and Supplementary Fig. 2 and 3). Interestingly, the 30x dataset has a higher recall for all four approaches. In addition, data with a higher sequencing depth (30x) have a lower precision compared to the sequencing data with a lower coverage (15x). The lower precision and higher recall of the 30x data could be attributed to higher number of misaligned reads leading to spurious SV calling[10].

To estimate the number of variants that are missed or incorrectly called using the 15x samples as compared to 30x, we obtained the true positives (TP), false positives (FP), and false negative (FN) counts for each pipeline across different sequencing depth. Across all SV pipelines, 15x libraries have a lower FP count compared to the 30x libraries (Supplementary Fig. 3). This could be attributed to the higher number of misaligned reads in the 30x libraries which could lead to spurious SV calls.

Supplementary Note 2: Benchmarking of SV calling pipeline for duplications

As the Manta-SVimmer-Graphtyper2 SV pipeline relies solely on discordant read pairs and split-read alignments, it has inherent limitations to accurately detect duplication events created by the presence of tandem repeat sequences (e.g., microsatellites and minisatellites)[11,12]. We thus complemented the above algorithms with SurVIndel2[13], an in-house developed algorithm that can detect duplication events at high sensitivity (Supplementary Fig. 4).

To demonstrate the robustness of SurVindel2, we assessed false discovery rate (FDR) and true positive (TP) statistics for duplications relative to Manta-SVimmer-Graphtyper2, against a truth set of high quality SVs obtained by haplotype-resolved long-read sequencing of a selected subset of 1000 Genomes Project analyzed samples[4].

We downloaded CRAM files at 30x coverage are available for all the samples[14]. We randomly selected 10 samples for our benchmarking effort and down-sampled the CRAM files to 15x using samtools[7] we ran our pipeline on a dataset comprising 5,487

discovery samples plus the 10 benchmarking samples. we ran our pipeline on a dataset comprising 5,487 discovery samples plus the 10 benchmarking samples. to mimic our discovery dataset.

For this benchmarking, we ran our pipeline on a dataset comprising 5,487 discovery samples plus the 10 benchmarking samples. we ran our pipeline on a dataset comprising 5,487 discovery samples plus the 10 benchmarking samples. Finally, we obtained a call set for each sample by retaining SVs with an allele count of at least 1 and an FS value of PASS.

We used an in-house tool (https://github.com/Mesh89/SVComparator) to compare, for each sample, the predicted SVs with the set of SVs reported in HGSVC2. Our pipeline reports tandem duplications and insertions separately, while HGSVC2 only reports deletions and insertions; tandem duplications are considered insertions. For this reason, we could not measure the sensitivity of our duplications and insertions separately.

We measured an average per-sample duplication identification FDR of 12% and 36% for SurVindel2 and Manta-SVimmer-Graphtyper2, respectively. SurVIndel2 yielded a better sensitivity than Manta-SVimmer-Graphtyper2 (Supplementary Fig. 3, Supplementary Table 2). Furthermore, the gains in sensitivity were more pronounced for tandem repeats (Supplementary Fig. 5).

One of the significant challenges when generating a dataset of SVs for a large population is maintaining a low level of noise. Our benchmarking efforts show that our call set is precise (average precision is 0.91 for deletions, 0.88 for duplications and 0.72 for insertions) (Supplementary Table 3). Unsurprisingly, long reads can discover far more SVs compared to 15x Illumina paired-end reads. However, the number of deletions, duplications and insertions we discovered is comparable to recent studies such as gnomAD[15] while using a lower sequencing depth. Coupled with the good precision, we conclude that our pipeline is in line with the state of the art in the field.

Supplementary Note 3: Identifying novel variants with respect to gnomAD-SV catalogue

To identify SVs that have a higher prevalence in Asian population within gnomAD-SV catalogue, we first identify variants that overlap between SG10K-SV and gnomAD-SV using SVimmer[5]. We identified 23,434 SVs in the SG10K-SV dataset which overlap with gnomAD-SV. This includes 4,725 deletions, 7,458 duplications and 11,251 insertions.

Supplementary Note 4: Identifying novel variants with respect to 1000G-SV catalogue

To identify SVs that have a higher prevalence in Asian population within 1000G-SV catalogue, we first identify variants that overlap between SG10K-SV and 1000G-SV using SVimmer[5]. We identified 9,668 SVs in the SG10K-SV dataset which overlap with 1000G-SV. This includes 3,105 deletions, 284 duplications and 6,279 insertions.

Supplementary Figures

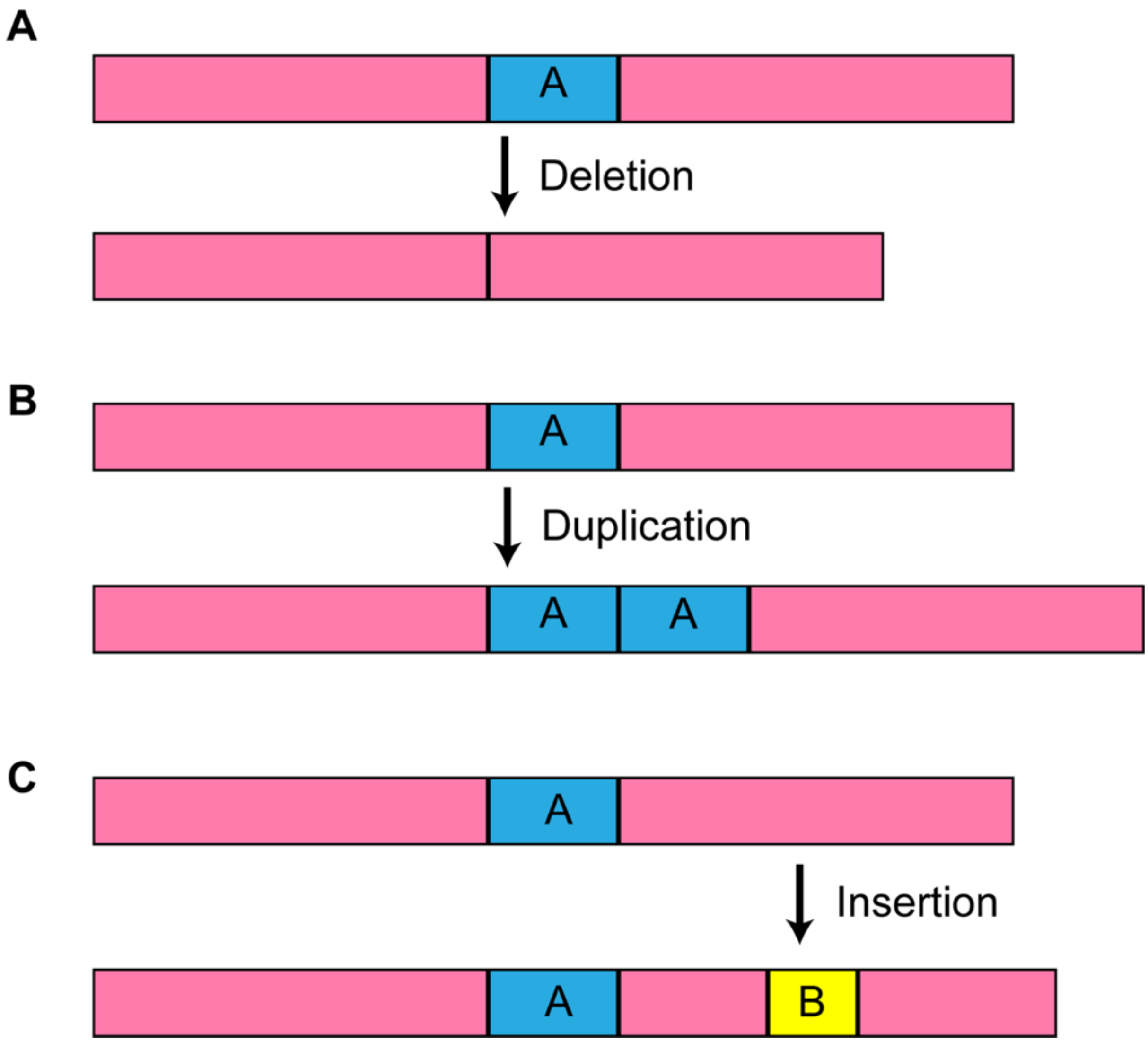

Supplementary Fig. 1 Different types of structural variations detected in SG10K-SV.

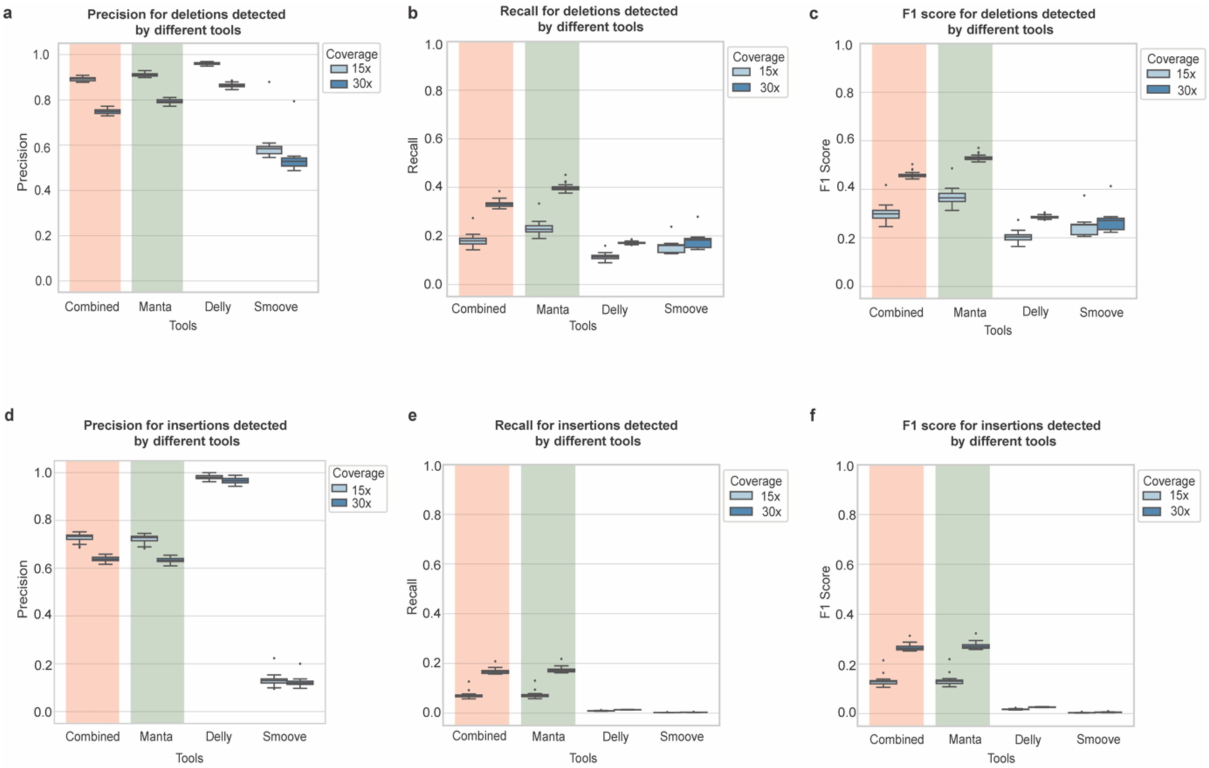

Supplementary Fig. 2 Benchmarking of various SV callers for deletions and insertions using 34 1000G samples with two different sequencing depths. **a** Boxplot showing the precision for deletions between 15x and 30x coverage for each caller. Combined refers to variants that are detected in all three pipelines. **b** Boxplot showing the recall for deletions between 15x and 30x coverage for each caller. **c** Boxplot showing the F1-score for deletions between 15x and 30x coverage for each caller. **d** Boxplot showing the precision for insertions between 15x and 30x coverage for each caller. **e** Boxplot showing the recall for insertions between 15x and 30x coverage for each caller. **f** Boxplot showing the F1-score for insertions between 15x and 30x coverage for each caller. The boxplots in a-f display the median and first/third quartiles.

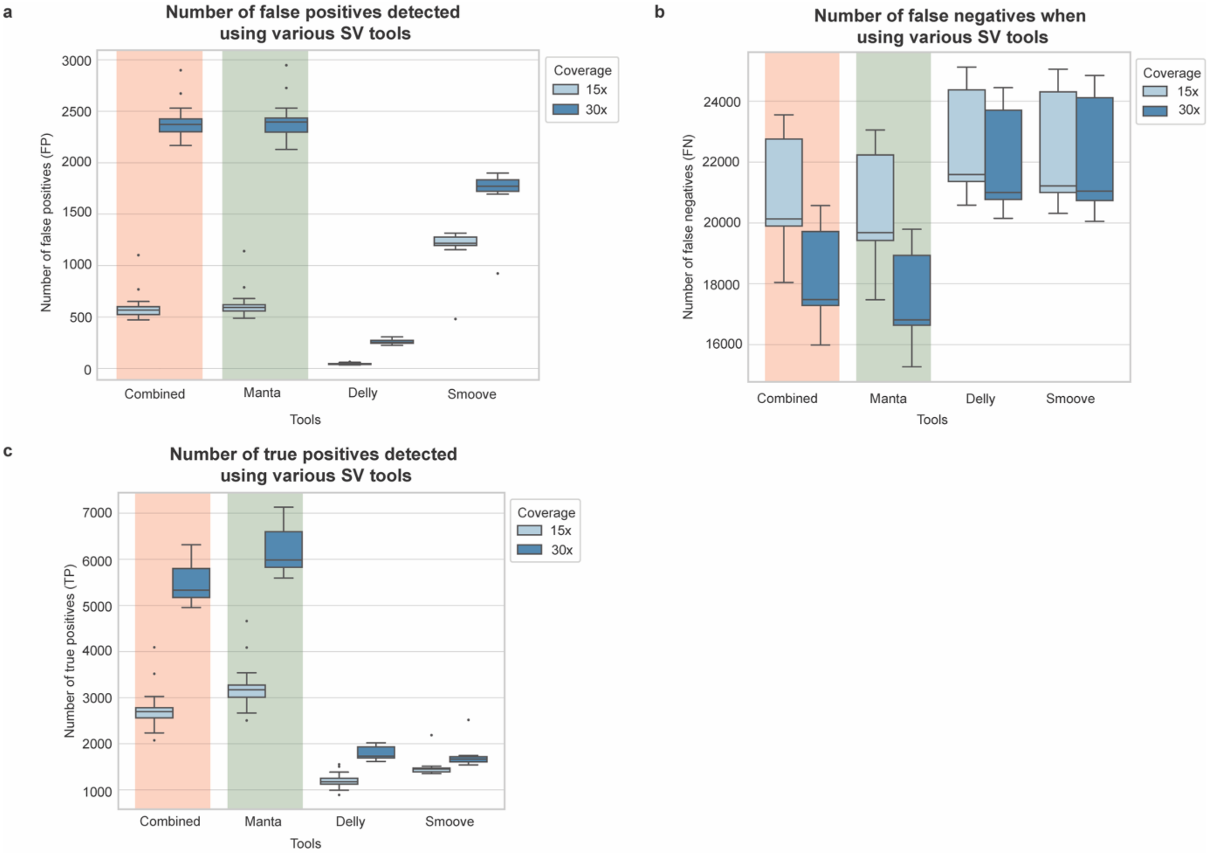

Supplementary Fig. 3 True positive, false positive and false negative counts for Manta, Delly, Smoove and their combination for all classed of SVs using 34 1000G samples with two different sequencing depth (15x and 30x coverage). **a** Boxplot showing the number of false positive counts between 15x and 30x coverage for each SV caller. Combined refers to variants that are detected in all three pipelines. **b** Boxplot showing the false negative counts between 15x and 30x coverage for each SV caller. **c** Boxplot showing the true positive counts between 15x and 30x coverage for each SV caller. The boxplots showed in a-c display the median and first/third quartiles.

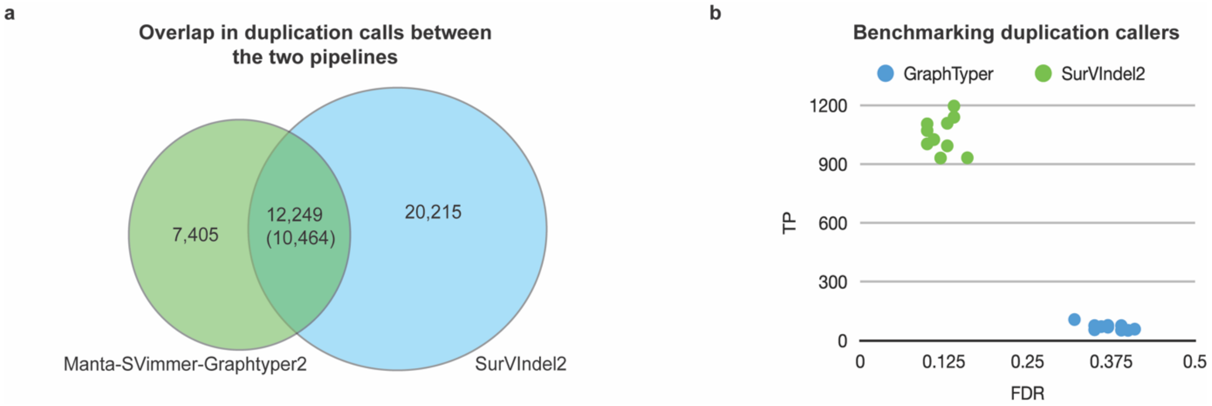

Supplementary Fig. 4 Comparison of SurVindel2 and Manta-SVimmer-Graphtyper2 pipeline for duplication identification.

a Comparison of the number of duplications detected by Manta-Graphtyper2 and SurVIndel2. **b** Scatterplot comparing the number of true positives detected duplication and FDR achieved with Manta-SVimmer-Graphtyper2 and SurVindel2 for a truth set of high quality SVs obtained by haplotype-resolved long-read sequencing of a selected subset of 1000 Genomes Project analyzed samples[4].

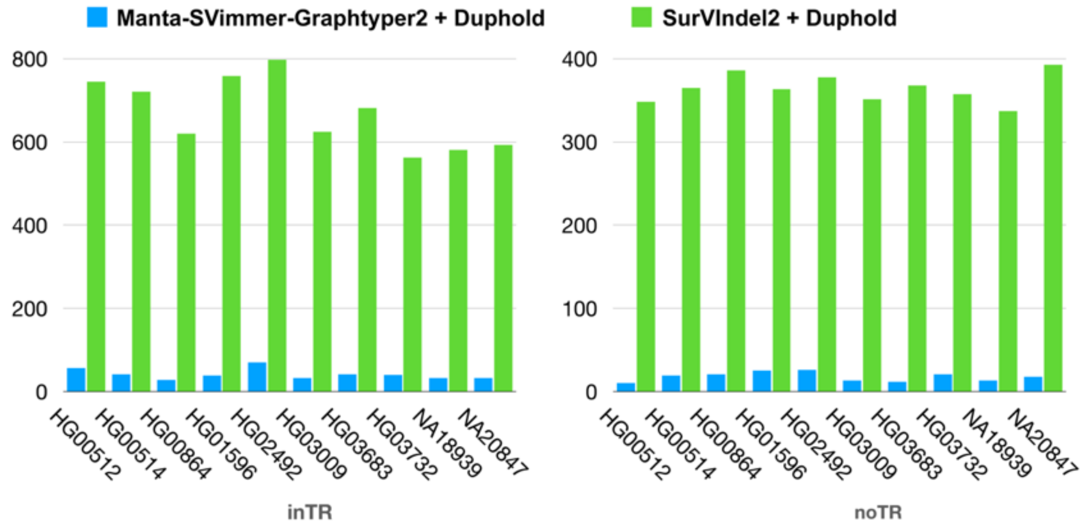

Supplementary Fig. 5 Barplot showing the number of duplications detected by Manta-SVimmer-Graphtyper2 and SurVindel2 in different genomic regions.
The Y-axis shows the number of SVs and the X-axis shows the sample name for each 1KG sample. Blue bars indicate the number of duplications detected in each 1KG sample by the Manta-SVimmer-Graphtyper2 pipeline. Green bars indicate the number of duplications detected in each 1KG sample by SurVindel2.
The barplot on the left shows the number of SVs detected by Manta-SVimmer-Graphtyper2 and SurVindel2 in tandem repeat regions. The barplot on the right shows the number of SVs Manta-SVimmer-Graphtyper2 and SurVindel2 in non-tandem repeat regions. SurVindel2 detects more duplications in both tandem repeat and non-tandem repeat regions compared to Manta-SVimmer-Graphtyper2.

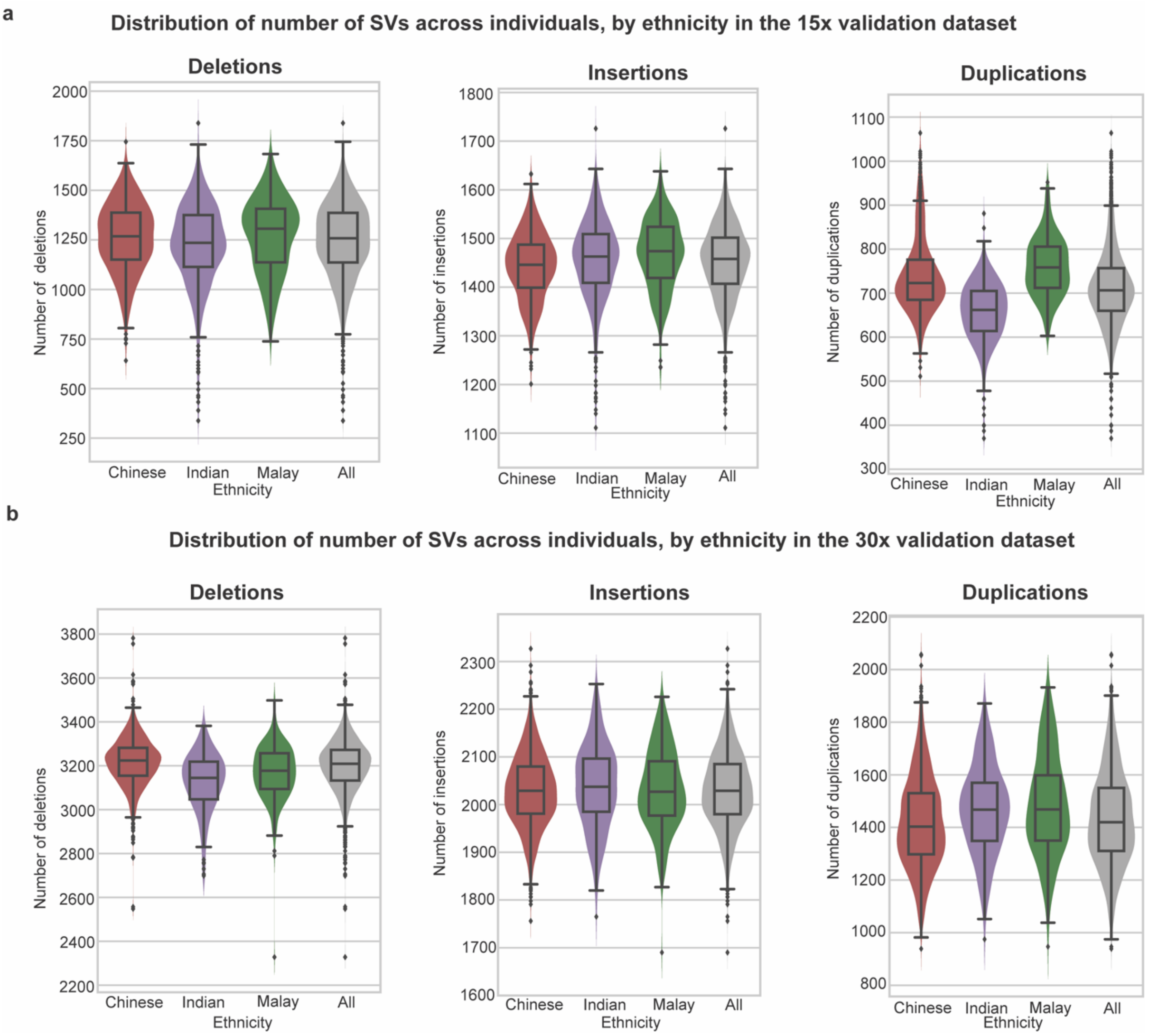

Supplementary Fig. 6 Violin plot showing the number of events per genome for the Validation datasets.

a Violin plots and boxplots showing the number of events per genome for each ethnic group (number of Chinese = 663, number of Malays = 278, number of Indians = 581). DEL, deletions; DUP, duplications; INS, insertions (including MEIs) in the 15x validation dataset. The boxplots display the minimum and maximum number of SVs as well as the median and the first/third quartile. **b** Violin plots and boxplots showing the number of events per genome for each ethnic group (number of Chinese = 1,433, number of Malays = 288, number of Indians = 198). DEL, deletions; DUP, duplications; INS, insertions (including MEIs) in the 30x validation dataset. The boxplots display the minimum and maximum number of SVs as well as the median and the first/third quartile.

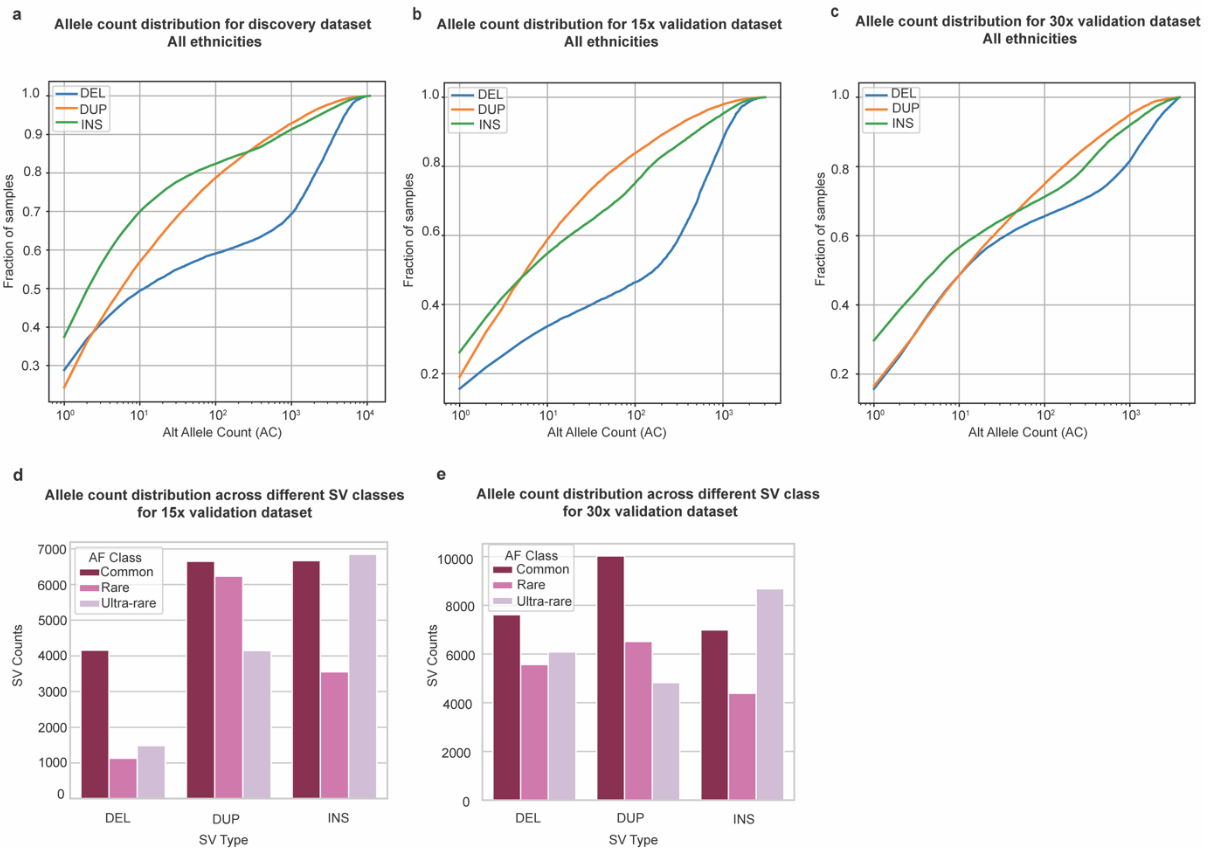

Supplementary Fig. 7 Allele distribution for the two validation datasets. **a** Distribution of alternate allele counts for different class of SVs in the discovery dataset. **b** Distribution of alternate allele counts for different classes of SVs in the SG10K 15x validation dataset. The majority of the SVs are rare variants (AF < 1%). **c** Distribution of alternate allele counts for different classes of SVs in the SG10K 30x validation dataset. The majority of the SVs are rare variants (AF < 1%). **d** Allele count distribution across different SV classes segregated by allele frequency classes for 15x validation dataset. Allele frequency (AF) bins: Common (AF ≥ 0.01), rare (0.01 > AF ≥ 0.001) and ultra-rare (AF < 0.001). **e** Allele count distribution across different SV classes segregated by allele frequency classes for 30x validation dataset.

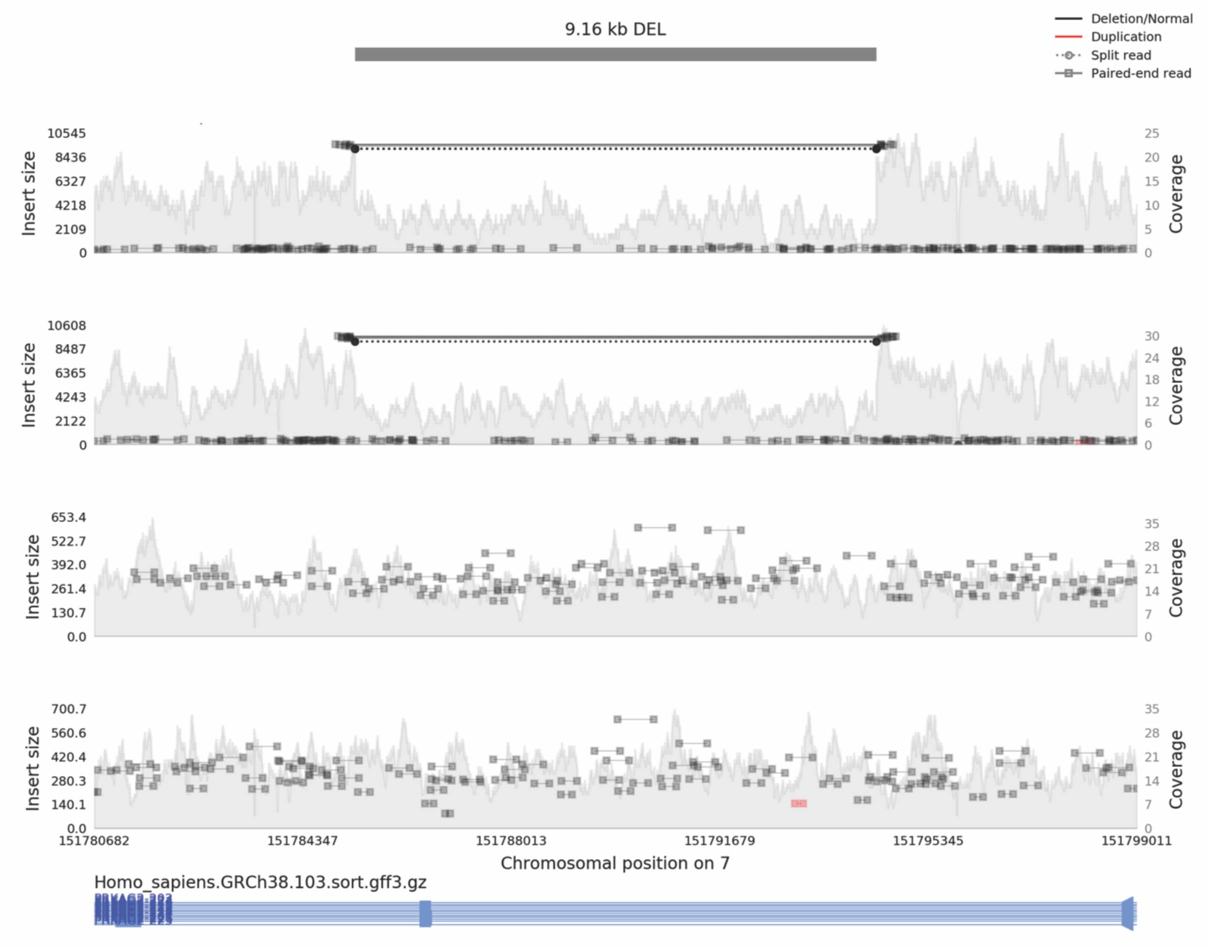

Supplementary Fig. 8 Samplot of a 9.16kb deletion event overlapping the *PRKAG2* gene region.

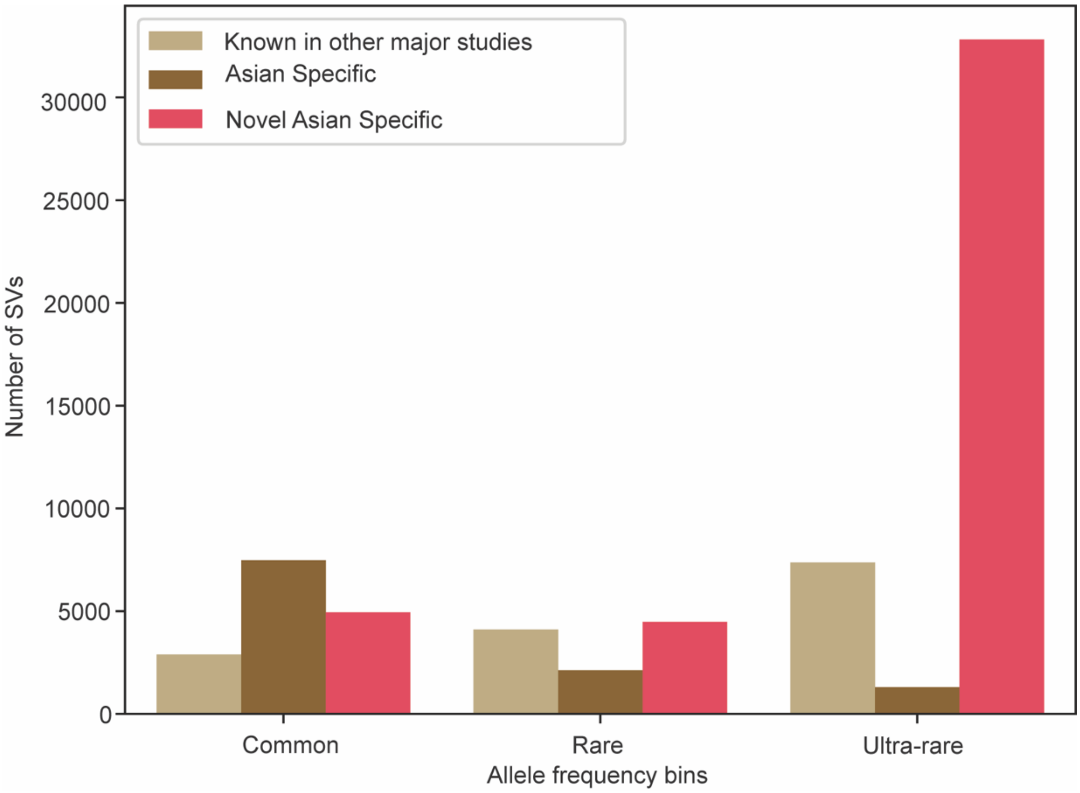

Supplementary Fig. 9: Distribution of novel Asian-specific and known Asian-specific SVs across different allele frequency bins.

Light-brown bars indicate SVs in SG10K-SV that overlap either gnomAD-SV or 1000G-SV and do not have significant Fst. Brown bars indicate SVs in SG10K-SV which overlap variants in either gnomAD-SV and 1000G and have significant Fst, and therefore, they are termed as "Asian-specific". Red bars indicate SVs that are only found in SG10K and have a call rate of ≥0.5 in Chinese, Malay, or Indians. These SVs are referred to as "Novel Asian Specific" SVs. The SVs are furthered partition into three different within SG10K-SV allele frequency (AF) bins: Common (AF ≥ 0.01), rare (0.01 > AF ≥ 0.001) and ultra-rare (AF < 0.001).

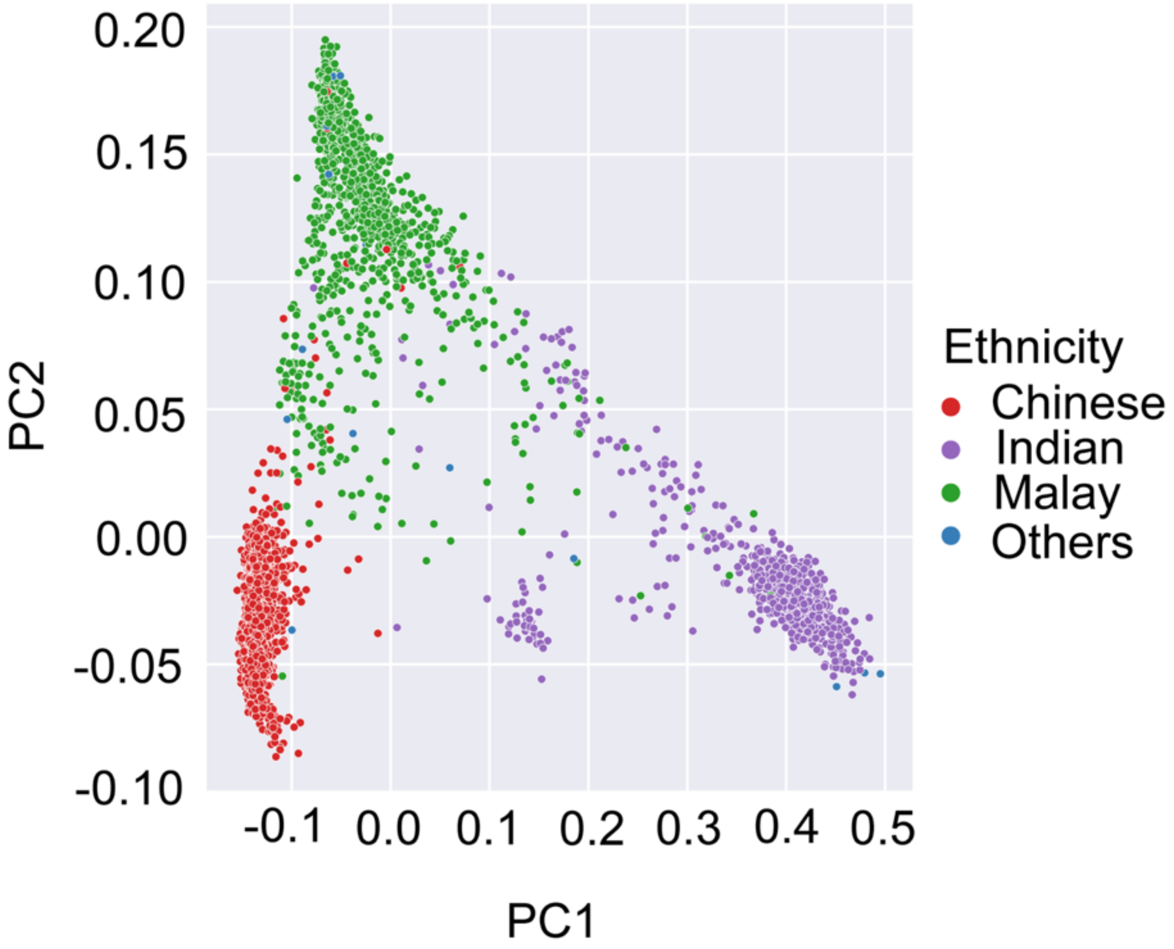

Supplementary Fig. 10 Scatter plot of the top-2 principal components of a SG10K_Health dataset Single Nucleotide Variant based PCA analysis showing the population structure in the Singaporean population.

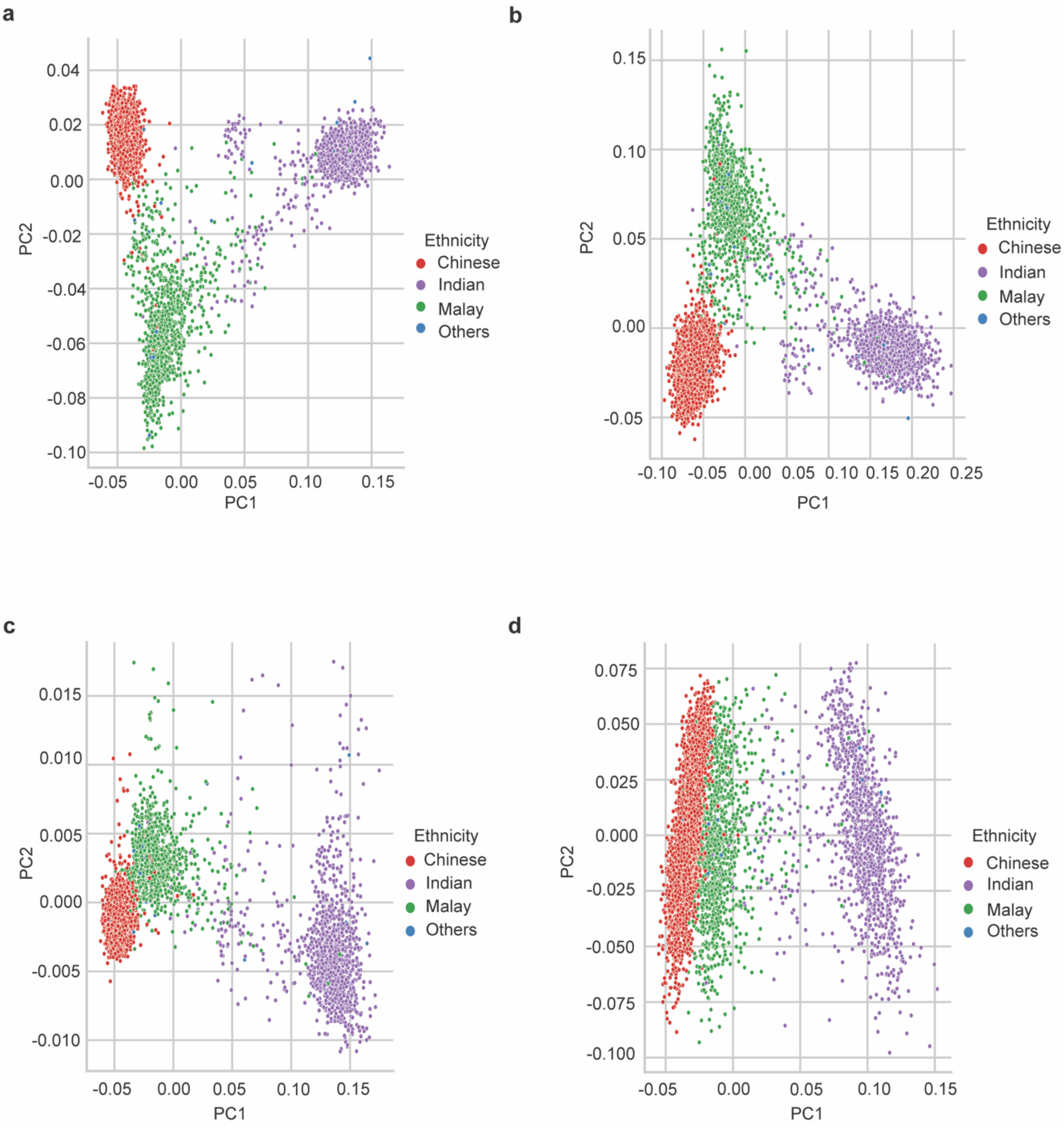

Supplementary Fig. 11 PCA of variants in the discovery dataset showing the population structure in the SG10K-SV-r1.4.
a PCA of all variants in the discovery dataset. **b** PCA using deletions only. **c** PCA using insertions only. **d** PCA using duplications only.

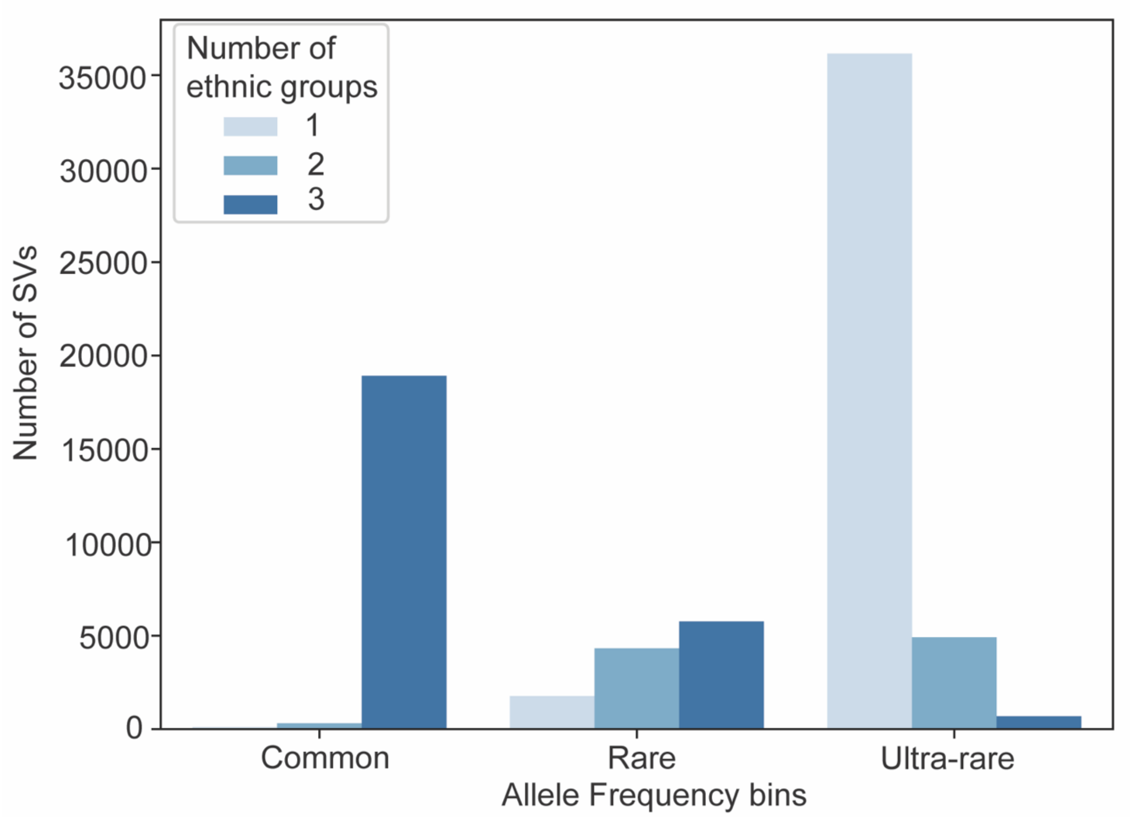

Supplementary Fig. 12 Distribution of SVs shared among ethnic group across different allele frequency bins.

Different shades of blue indicate the number of ethnic groups in which the SV is detected in. The SVs are furthered partition into three different allele frequency bins. Common indicates variants with allele frequency ≥ 0.01; rare indicates variants with allele frequency ≥ 0.001 and allele frequency < 0.01; ultra-rare variants refers to variants with allele frequency < 0.001.

Supplementary references

1. Chen, X. *et al.* Manta: rapid detection of structural variants and indels for germline and cancer sequencing applications. *Bioinformatics* **32**, 1220-2 (2016).
2. Rausch, T. *et al.* DELLY: structural variant discovery by integrated paired-end and split-read analysis. *Bioinformatics* **28**, i333-i339 (2012).
3. Pedersen, B.S., Layer, R., Quinlan, A. R. smoove: structural-variant calling and genotyping with existing tools. 0.2.8 edn (https://github.com/brentp/smoove, 2020).
4. Ebert, P. *et al.* Haplotype-resolved diverse human genomes and integrated analysis of structural variation. *Science* **372**(2021).
5. Eggertsson, H.P. Structural Variant Merging Tool. (https://github.com/DecodeGenetics/svimmer, 2021).
6. Eggertsson, H.P. *et al.* GraphTyper2 enables population-scale genotyping of structural variation using pangenome graphs. *Nature Communications* **10**, 5402 (2019).
7. Danecek, P. *et al.* Twelve years of SAMtools and BCFtools. *GigaScience* **10**(2021).
8. English, A.C., Menon, V.K., Gibbs, R.A., Metcalf, G.A. & Sedlazeck, F.J. Truvari: refined structural variant comparison preserves allelic diversity. *Genome Biology* **23**, 271 (2022).
9. Tarasov, A., Vilella, A.J., Cuppen, E., Nijman, I.J. & Prins, P. Sambamba: fast processing of NGS alignment formats. *Bioinformatics* **31**, 2032-2034 (2015).
10. Kosugi, S. *et al.* Comprehensive evaluation of structural variation detection algorithms for whole genome sequencing. *Genome Biology* **20**, 117 (2019).
11. Rajaby, R. & Sung, W.K. SurVIndel: improving CNV calling from high-throughput sequencing data through statistical testing. *Bioinformatics* **37**, 1497-1505 (2021).
12. Dashnow, H. *et al.* STRetch: detecting and discovering pathogenic short tandem repeat expansions. *Genome Biology* **19**, 121 (2018).
13. Rajaby, R. & Sung, W.-K. SurVIndel2: improving local CNVs calling from next-generation sequencing using novel hidden information. *bioRxiv*, 2023.04.23.538018 (2023).
14. Byrska-Bishop, M. *et al.* High-coverage whole-genome sequencing of the expanded 1000 Genomes Project cohort including 602 trios. *Cell* **185**, 3426-3440.e19 (2022).
15. Abel, H.J. *et al.* Mapping and characterization of structural variation in 17,795 human genomes. *Nature* **583**, 83-89 (2020).

SG10K_Health Consortium

Khung Keong Yeo[34], Stuart Alexander Cook[34], Chee Jian Pua[34], Chengxi Yang[34], Tien Yin Wong[12], Charumathi Sabanayagam[12,35], Lavanya Raghavan[12], Tin Aung[12,35], Miao Ling Chee[12], Miao Li Chee[12], Hengtong Li[12,13], Jimmy Lee[36,37], Eng Sing Lee[38,39], Joanne Ngeow[23,40], Paul Eillot[41], Elio Riboli[41], Hong Kiat Ng[23], Theresia Mina[23], Darwin Tay[23], Nilanjana Sadhu[23], Pritesh Rajesh Jain[23], Dorrain Low[23], Xiaoyan Wang[23], Jin Fang Chai[7], Rob M Van Dam[7,42,43], Yik Ying Teo[7], Chia Wei Lim[11], Pi Kuang Tsai[11], Wen Jie Chew[44], Wey Ching Sim[11], Li-xian Grace Toh[11], Johan Gunnar Eriksson[19,45], Peter D Gluckman[46,47], Yung Seng Lee[19,48], Fabian Yap[49], Kok Hian Tan[50]

[1]Genome Institute of Singapore, Agency for Science, Technology and Research, Singapore
[2]Duke-NUS Medical School, Singapore
[3]SingHealth Duke-NUS Institute of Precision Medicine, Singapore Health Services, Duke-NUS Medical School, Singapore
[4]SingHealth Duke-NUS Genomic Medicine Centre, Duke-NUS Medical School, Singapore
[5]Saw Swee Hock School of Public Health, National University of Singapore and National University Health System, Singapore
[6]Department of Obstetrics & Gynaecology, Yong Loo Lin School of Medicine, National University of Singapore, Singapore
[7]Institute for Human Development and Potential (IHDP), Agency for Science, Technology and Research (A*STAR), Singapore
[8]NHG Eye Institute, Tan Tock Seng Hospital, National Healthcare Group, Singapore
[9]Personalised Medicine Service, Tan Tock Seng Hospital, Singapore
[10]Singapore Eye Research Institute, Singapore National Eye Centre, Singapore
[11]Centre for Innovation and Precision Eye Health, Yong Loo Lin School of Medicine, National University of Singapore, Singapore
[12]Department of Cardiology, National Heart Centre Singapore, Singapore
[13]Cardiovascular ACP, Duke-NUS Medical School, Singapore
[14]SingHealth Duke-NUS Institute of Precision medicine, Singapore Health Services, Singapore
[15]Cardiovascular and Metabolic Disorders Program, Duke-NUS Medical School, Singapore
[16]Current affiliation: Translational Medicine, Sidra Medicine, Qatar
[17]Human Development, Singapore Institute for Clinical Sciences, Singapore
[18]Clinical Data Engagement, Bioinformatics Institute, Agency for Science, Technology and Research, Singapore
[19]Department of Biochemistry, Yong Loo Lin School of Medicine, National University of Singapore, Singapore
[20]Centre for Innovation and Precision Eye Health, Yong Loo Lin School of Medicine, National University of Singapore, Singapore
[21]Population and Global Health, Nanyang Technological University, Lee Kong Chian School of Medicine, Singapore
[22]Department of Epidemiology and Biostatistics, Imperial College London, UK
[23]Precision Health Research, Singapore
[24]Department of Medicine, Yong Loo Lin School of Medicine, National University of Singapore, Singapore

[25]Saw Swee Hock School of Public Health, National University of Singapore and National University Health System, Singapore
[26]Laboratory of Human Genomics, Genome Institute of Singapore (GIS), Agency for Science, Technology and Research (A*STAR), Singapore
[27]Yong Loo Lin School of Medicine, National University of Singapore, Singapore
[28]Lee Kong Chian School of Medicine, Nanyang Technological University, Singapore
[29]Cancer Science Institute of Singapore, National University of Singapore, Singapore
[30]National Heart Research Institute Singapore, National Heart Centre Singapore, Singapore
[31]Ophthalmology & Visual Sciences Academic Clinical Program, Duke-NUS Medical School, Singapore
[32]Department of Psychosis, Institute of Mental Health, Singapore
[33]Nanyang Technological University, Singapore
[34]National Healthcare Group, Singapore
[35]Nanyang Technological University, Lee Kong Chian School of Medicine, Singapore
[36]National Cancer Centre, Singapore
[37]School of Public Health, Imperial College London, UK
[38]Department of Nutrition, Harvard T.H. Chan School of Public Health, US
[39]Exercise and Nutrition Sciences, Milken Institute School of Public Health, The George Washington University, US
[40]Clinical Research & Innovation Office, Tan Tock Seng Hospital, Singapore,
[41]Department of Obstetrics & Gynaecology, Yong Loo Lin School of Medicine, NUS, Singapore
[42]Singapore Institute for Clinical Sciences, Singapore
[43]Liggins Institute, University of Auckland, New Zealand
[44]Department of Paediatrics, National University Hospital, Singapore
[45]Pediatrics, KK Women's and Children's Hospital, Singapore
[46]Department of Obstetrics & Gynaecology, KK Women's and Children's Hospital, Singapore
