# Supplementary material for: A Catalogue of Structural Variation across Ancestrally Diverse Asian Genomes
Source: Nat Commun. 2024 Nov 4;15:9507. doi: 10.1038/s41467-024-53620-8 (PMC11535549; doi:10.1038/s41467-024-53620-8)
Supplement: Supplementary file 3 — Description of Additional Supplementary Files [file 41467_2024_53620_MOESM3_ESM.pdf]

Description of Additional Supplementary Files

File Name: Supplementary Data 1
Description: Demographics of SG10K samples across different ethnic groups

File Name: Supplementary Data 2
Description: Precision and Recall of deletions using different SV calling pipelines using 34 1000G samples with corresponding long read data.

File Name: Supplementary Data 3
Description: Benchmarking duplications in ten 1KG samples using the SG10K-SV workflow

File Name: Supplementary Data 4
Description: Sensitivity and Precision of our pipeline measured using ten 1000Genome project samples

File Name: Supplementary Data 5
Description: Hotspot location detected using the SG10K-SV variants.

File Name: Supplementary Data 6
Description: Distribution of SVs disrupting regulatory regions (ENCODE cCREs) across allele frequency bins.

File Name: Supplementary Data 7
Description: Distribution of SVs disrupting gene centric features across allele frequency bins.

File Name: Supplementary Data 8
Description: Selected 86 events which shows signficant Fst between ethnic groups.

File Name: Supplementary Data 9
Description: Linkage Disequilibrium R2 between SV and lead GWAS SNP obtain from the GWAS Catalog. The P-values were obtained from each individual study.
