# Supplementary material for: A Catalogue of Structural Variation across Ancestrally Diverse Asian Genomes
Source: Nat Commun. 2024 Nov 4;15:9507. doi: 10.1038/s41467-024-53620-8 (PMC11535549; doi:10.1038/s41467-024-53620-8)
Supplement: Supplementary file 5 — Reporting Summary [file 41467_2024_53620_MOESM5_ESM.pdf]

nature portfolio

Corresponding author(s): Nicolas Bertin, Patrick Tan, Shyam Prabhakar

Last updated by author(s): Sep 6, 2024

Reporting Summary

Nature Portfolio wishes to improve the reproducibility of the work that we publish. This form provides structure for consistency and transparency in reporting. For further information on Nature Portfolio policies, see our Editorial Policies and the Editorial Policy Checklist.

Statistics

For all statistical analyses, confirm that the following items are present in the figure legend, table legend, main text, or Methods section.

n/a	Confirmed	
☐	☒	The exact sample size (*n*) for each experimental group/condition, given as a discrete number and unit of measurement
☐	☒	A statement on whether measurements were taken from distinct samples or whether the same sample was measured repeatedly
☐	☒	The statistical test(s) used AND whether they are one- or two-sided<br>*Only common tests should be described solely by name; describe more complex techniques in the Methods section.*
☐	☒	A description of all covariates tested
☐	☒	A description of any assumptions or corrections, such as tests of normality and adjustment for multiple comparisons
☐	☒	A full description of the statistical parameters including central tendency (e.g. means) or other basic estimates (e.g. regression coefficient) AND variation (e.g. standard deviation) or associated estimates of uncertainty (e.g. confidence intervals)
☐	☒	For null hypothesis testing, the test statistic (e.g. *F*, *t*, *r*) with confidence intervals, effect sizes, degrees of freedom and *P* value noted<br>*Give P values as exact values whenever suitable.*
☒	☐	For Bayesian analysis, information on the choice of priors and Markov chain Monte Carlo settings
☒	☐	For hierarchical and complex designs, identification of the appropriate level for tests and full reporting of outcomes
☐	☒	Estimates of effect sizes (e.g. Cohen's *d*, Pearson's *r*), indicating how they were calculated

Our web collection on statistics for biologists contains articles on many of the points above.

Software and code

Policy information about availability of computer code

Data collection	Each sample is sequenced on Illumina HiSeq X. Raw data are processed using home-made nextflow workflow running on singularity or docker container. Trimming, mapping, duplicates detection, variant calling, variant recalibration and QC are performed following GATK "germline short variant per-sample calling" Reference Implementation defined parameters and companion files (GATK resource bundle GRCh38). see software requirements - awscli v1.15.53, bcftools v1.8, bwa v0.7.17, datamash v1.3, fastqc v0.11.7, freebayes v1.2.0, gatk4 v4.0.6.0, goleft v0.1.18, picard v2.18.9, samtools v1.8, seqtk v1.3, trimadap r11, verifybamid=1.1.3
Data analysis	Deletions and insertions were detected using Manta in single samples, followed by SVimmer to obtain a putative cohort-wide consensus set. Individual-level genotypes were then refined using Graphtyper2. Mobile element insertions were detected using MELT. Duplications were detected using SurVindel2. Downstream analysis was performed on Hail. LD between SNPs and SVs was computed using Plink v1.9. All codes used for the study are available on Github (https://github.com/c-BIG/SG10K-SV-MANUSCRIPT)

For manuscripts utilizing custom algorithms or software that are central to the research but not yet described in published literature, software must be made available to editors and reviewers. We strongly encourage code deposition in a community repository (e.g. GitHub). See the Nature Portfolio guidelines for submitting code & software for further information.

Data

Policy information about availability of data

All manuscripts must include a data availability statement. This statement should provide the following information, where applicable:

- Accession codes, unique identifiers, or web links for publicly available datasets
- A description of any restrictions on data availability
- For clinical datasets or third party data, please ensure that the statement adheres to our policy

The SG10K-SV aggregated SV callset will be made available in the CHORUS browser through the SG10K_Health web portal (https://npm.a-star.edu.sg/). Registered users will be able to download the aggregated SV calls. Efforts were also undertaken to establish a robust and transparent process to enable access to the genomic sequence data through the NPM Data Access Committee (DAC) via (contact_npco@gis.a-star.edu.sg).

Research involving human participants, their data, or biological material

Policy information about studies with human participants or human data. See also policy information about sex, gender (identity/presentation), and sexual orientation and race, ethnicity and racism.

Reporting on sex and gender	The SG10K-SV dataset contains a subset of the original SG10K_HEALTH Dataset.<br>Following samples (n=8,392) used to generate Supplementary Table 1.<br>Sex: 3,746 male and 5,186 females
Reporting on race, ethnicity, or other socially relevant groupings	The SG10K-SV dataset contains a subset of the original SG10K_HEALTH Dataset.<br>Following samples (n=8,392) used to generate Supplementary Table 1.<br>Genotypic ancestry: East Asians, South Asians, Southeast Asians, Others<br>Self-reported ethnicity: 5,184 Chinese, 2,106 Indian, 1,620 Malay and 22 Others
Population characteristics	The SG10K-SV dataset contains a subset of the original SG10K_HEALTH Dataset.<br>Following samples (n=8,392) used to generate Supplementary Table 1.<br>Sample count by cohort: MEC: 2,794, HELIOS: 1,779, SEED: 1,436, GUSTO: 969, PRISM: 1,040, TTSH: 914<br>All participants were healthy at the point of recruitment
Recruitment	The SG10K_Health dataset is aggregated from six prospective cohorts: MEC, HELIOS, SEED, GUSTO, PRISM, and TTSH. Participants were recruited by the participating cohorts. Each recruited individual or parent/guardian, in the case of minors, signed an informed consent with the participating cohort according to the respective study protocols.<br><br>MEC cohort includes Singapore citizens or long-term residents aged 21 to 75 years. Individuals with a history of heart disease, stroke, cancer, and renal failure were excluded at baseline. Participants were recruited from two existing population-based studies Singapore Prospective Study Program (SP2) and the Singapore Cardiovascular Cohort Study (SCCS2), public outreach programs, and referrals from existing cohort members. Written consent was obtained for registry and medical records linkage, future analysis of stored biological samples, and future follow-up. (Tan KHX et al. Cohort Profile: The Singapore Multi-Ethnic Cohort (MEC) study. Int J Epidemiol 2018; 47:699-699)<br><br>HELIOS cohort includes Singapore citizens or Permanent Residents aged 30-84 years old and excludes pregnant and breastfeeding women, those with major illnesses requiring hospitalization /surgery, cancer treatment in the past year, or participating in drug trials within the past month. Participants were recruited from the general population through community outreach programs to ensure diversity in ethnicity and socio-economic background. (www.healthforlife.sg)<br><br>SEED cohort is based on an age-stratified random sampling strategy of individuals between the ages of 40 and 80+ years from 15 residential districts in the South-Western part of Singapore. (Majithia S et al. Cohort Profile: The Singapore Epidemiology of Eye Diseases (SEED) study. Int J Epidemiol 2021: 50:41-52)<br><br>GUSTO study recruited pregnant women aged 18 years and above, attending their first-trimester antenatal dating ultrasound scan clinic at Singapore's two major public maternity units, National University Hospital (NUH) and KK Women's and Children's Hospital (KKH), between June 2009 and September 2010. The participants approached were Singapore citizens or permanent residents who were of Chinese, Malay or Indian ethnicity with homogeneous parental ethnic background and who had the intention of eventually delivering in NUH or KKH and residing in Singapore for the next 5 years. Only women who agreed to donate birth tissues including cord, placenta and cord blood at delivery were included. Informed written consent was obtained from each participant. Of the 1247 women recruited, 1162 conceived naturally and 85 conceived through in vitro fertilisation (IVF). A total of 1176 babies were born and longitudinally assessed on their growth and development. (Soh SE et al. Cohort Profile: Growing Up in Singapore Towards healthy Outcomes (GUSTO) birth cohort study. Int J Epidemiol 2014 Oct:43(5):1401-9)<br><br>PRISM cohort includes healthy volunteers aged over 18 years old from the Singapore population with no known pre-existing health conditions. Participants were recruited through an advertisement in a local daily newspaper and consented to the collection of demographic, personal, and family history as well as medical records to be accessed. (Bylstra Y et al. Implementation of genomics in medical practice to deliver precision medicine for an Asian population. npj Genom Med 2019;4-1-7)

	TTSH cohort recruited 4,000 participants in the Tan Tock Seng Hospital Healthy Control Registry. The inclusion criteria were: ability to give informed consent, age 21 and older, do not have any personal history of major disorders such as stroke, cardiovascular diseases, cancer, diabetes, renal failure. The participants were recruited mainly from health screening exercises carried out in the hospital and the community. After the participants had provided written consent, biographic data, medical information, and blood were collected. All identifiable data have been irreversibly delinked from the collection. (https://www.ttsh.com.sg/Patients-and-Visitors/Medical-Services/personalised-medicine/Pages/default.aspx)
Ethics oversight	All participants have provided informed consent for research. IRB approvals from each participating cohort were obtained from their respective contributing organizations: MEC cohort (National University of Singapore IRB, B-16-158), HELIOS (Nanyang Technological University IRB, 2016-11-030 and 2017-11-006-01), SEED (SingHealth Centralised Institutional Review Board, 2012/487/A, 2010/392/A and 2015/2279), GUSTO (SingHealth Centralised Institutional Review Board, 2018/2767 and National Health Group Domain Specific Review Board, D/2009/00021 and B/2014/00406), PRISM (SingHealth Centralised Institutional Review Board, 2013/605/C) and TTSH (National Health Group R&D office, TTSH/2014-00040).

Note that full information on the approval of the study protocol must also be provided in the manuscript.

Field-specific reporting

Please select the one below that is the best fit for your research. If you are not sure, read the appropriate sections before making your selection.

☒ Life sciences ☐ Behavioural & social sciences ☐ Ecological, evolutionary & environmental sciences

For a reference copy of the document with all sections, see nature.com/documents/nr-reporting-summary-flat.pdf

Life sciences study design

All studies must disclose on these points even when the disclosure is negative.

Sample size	NPM Phase I aim to generate a genomic reference database of 10,000 healthy Singaporeans (SG10K_Health). The sample size (n=8,392) after filtering achieved Chinese 58%, Indian 23.6%, Malay 18.1% and Others 0.3%, provided a starting catalog of structural variations in the three major ethnic groups.
Data exclusions	The paired-end reads were adaptor-trimmed using Trimadap and mapped using BWA-MEM to GRCh38 reference. Duplicated reads were discarded using Picard MarkDuplicates with GATK "germline short variant per-sample calling" Reference Implementation defined parameters. Base quality scores were recalibrated using GATK BaseRecalibrator / ApplyBQSR on a per library basis.<br>Sex was imputed based on the mean depth ratio of chrX/chr20 and chrY/chr20 of each sample, and samples with abnormal ploidy were also excluded. Subsequently, samples with call rate < 95%, contamination rate > 2%, and error rate > 1.5% were also excluded.<br>Samples with a deviation of more than 6x the Median Average Deviation (MAD) for autosome SNPs' insertion/deletion, transition/transversion, and heterozygote/homozygote ratios were excluded, giving 9,770 healthy individuals in the SG10K_Health dataset.<br>In addition, we calculated nine different metrics such as median autosome coverage, MAD autosome coverage, percentage of bases that attained at least 1X sequence coverage in autosomes, percentage of PF (pass filter) reads that align to the reference, percentage of reads that align as proper pairs as calculated with samtools stats, median insert size of aligned reads, MAD of insert sizes, Illumina-style GC dropout metric, Illumina-style AT dropout metric. In each cohort, we discarded samples that fall outside 8 MAD from the median for at least one of the nine metrics. These filters led to the exclusion of 1,378 samples, thus leaving 8,392 samples for downstream analysis.
Replication	The study aim is to sequence as many samples to achieve 10,000 genomes the work does not include replication study.
Randomization	This is not an experimental study so randomization is not required.
Blinding	This is not an experimental study so blinding is not required.

Reporting for specific materials, systems and methods

We require information from authors about some types of materials, experimental systems and methods used in many studies. Here, indicate whether each material, system or method listed is relevant to your study. If you are not sure if a list item applies to your research, read the appropriate section before selecting a response.

Materials & experimental systems

n/a	Involved in the study
☒	☐ Antibodies
☒	☐ Eukaryotic cell lines
☒	☐ Palaeontology and archaeology
☒	☐ Animals and other organisms
☒	☐ Clinical data
☒	☐ Dual use research of concern
☒	☐ Plants

Methods

n/a	Involved in the study
☒	☐ ChIP-seq
☒	☐ Flow cytometry
☒	☐ MRI-based neuroimaging

Plants

Seed stocks	*Report on the source of all seed stocks or other plant material used. If applicable, state the seed stock centre and catalogue number. If plant specimens were collected from the field, describe the collection location, date and sampling procedures.*
Novel plant genotypes	*Describe the methods by which all novel plant genotypes were produced. This includes those generated by transgenic approaches, gene editing, chemical/radiation-based mutagenesis and hybridization. For transgenic lines, describe the transformation method, the number of independent lines analyzed and the generation upon which experiments were performed. For gene-edited lines, describe the editor used, the endogenous sequence targeted for editing, the targeting guide RNA sequence (if applicable) and how the editor was applied.*
Authentication	*Describe any authentication procedures for each seed stock used or novel genotype generated. Describe any experiments used to assess the effect of a mutation and, where applicable, how potential secondary effects (e.g. second site T-DNA insertions, mosiacism, off-target gene editing) were examined.*
